# Bioprocessing strategies to enhance the challenging isolation of neuro-regenerative cells from olfactory mucosa

**DOI:** 10.1038/s41598-018-32748-w

**Published:** 2018-09-27

**Authors:** Melanie Georgiou, Joana Neves dos Reis, Rachael Wood, Patricia Perez Esteban, Victoria Roberton, Chris Mason, Daqing Li, Ying Li, David Choi, Ivan Wall

**Affiliations:** 1 Department of Biochemical Engineering, University College London, Torrington Place, London, WC1E 7JE UK; 2 Present Address: Cell and Gene Therapy Catapult, Guy’s Hospital, Great Maze Pond, London, SE1 9RT UK; 3 Aston Medical Research Institute and School of Life & Health Sciences, Aston University, Aston Triangle, Birmingham, B4 7ET UK; 4 Spinal Repair Unit, Department of Brain, Repair and Rehabilitation, Institute of Neurology, University College London, Queen Square, London, WC1N 3BG UK; 5 National Hospital for Neurology and Neurosurgery, Queen Square, London, WC1N 3BG UK; 6 Institute of Tissue Regeneration Engineering (ITREN), Dankook University, Cheonan, 31116 Republic of Korea

## Abstract

Olfactory ensheathing cells (OECs) are a promising potential cell therapy to aid regeneration. However, there are significant challenges in isolating and characterizing them. In the current study, we have explored methods to enhance the recovery of cells expressing OEC marker p75NTR from rat mucosa. With the addition of a 24-hour differential adhesion step, the expression of p75NTR was significantly increased to 73 ± 5% and 46 ± 18% on PDL and laminin matrices respectively. Additionally, the introduction of neurotrophic factor NT-3 and the decrease in serum concentration to 2% FBS resulted in enrichment of OECs, with p75NTR at nearly 100% (100 ± 0% and 98 ± 2% on PDL and laminin respectively), and candidate fibroblast marker Thy1.1 decreased to zero. Culturing OECs at physiologically relevant oxygen tension (2–8%) had a negative impact on p75NTR expression and overall cell survival. Regarding cell potency, co-culture of OECs with NG108-15 neurons resulted in more neuronal growth and potential migration at atmospheric oxygen. Moreover, OECs behaved similarly to a Schwann cell line positive control. In conclusion, this work identified key bioprocessing fundamentals that will underpin future development of OEC-based cell therapies for potential use in spinal cord injury repair. However, there is still much work to do to create optimized isolation methods.

## Introduction

Regeneration within the central nervous system (CNS) generally does not occur naturally. On the other hand, the olfactory system is characterized by its ability for sensory neuron regeneration throughout life in healthy humans and animals following injury or disease. Olfactory ensheathing cells (OECs), the glial cells of the olfactory system, play a key role to support the regeneration and guidance of olfactory receptor neurons from the peripheral nervous system into the central nervous system by creating a permissive environment for neurite outgrowth^1–4^. Due to this unique ability, OECs have been investigated extensively over the years for use in a potential spinal cord repair cell therapy, where spontaneous regeneration does not occur after injury and so surgical intervention is required^5–7^.

OECs are located in the olfactory bulb in the brain and the olfactory mucosa within the nasal cavity^8,9^. From a clinical perspective, mucosa-derived OECs are a more attractive source because they can be more easily accessed via a minimally invasive intranasal approach, avoiding the more complicated intracranial approach to obtain bulbar cells. However, there are many challenges associated with using mucosal biopsies; mainly the relatively small yield and purity of OECs obtained. Using present protocols, the purity from mucosal biopsies is less than 5%, compared with around 50% from bulbar biopsies^10^, and therefore the majority of studies use bulb-derived OECs^11–15^. Currently, literature is divided regarding an isolation method that is able to effectively purify the OECs from other cell types (i.e. olfactory fibroblasts and other accessory cells); hence transplantation of OECs for neural repair typically contains a mixed glial population resulting in variation in the derived cell populations from each cell preparation. In turn, this cell heterogeneity causes variability in treatment outcomes. The isolation and culture method has also been shown to affect the efficacy of the OECs to support spinal cord regeneration^16^.

Transplantation of OECs into the injured spinal cord has shown positive therapeutic effects in animal models^13,17^ and in human phase I clinical trials, demonstrating the safety of OEC transplantation^18,19^. However, the results from *in vivo* studies are variable, and in some cases no anatomical improvements or functional recovery are evident^20–24^.

Work has gone some way to optimize the isolation and culture methods for OECs; however, these have mainly been for bulb-derived OECs^5,25–27^. Studies have investigated the effect of serum concentration, or the addition of neurotrophic factors amongst other variables on the isolation and culture of mucosal OECs, but a full characterization of these cells is yet to be established in the literature. Here we aim to investigate how bioprocess modifications, selected based on previous reports in the literature, to the commonly used isolation method can affect the resulting cell population from rat olfactory mucosal tissue, in terms of their expression of OEC markers (p75NTR), glial cell markers (GFAP, S100β), neural precursor markers (nestin and βIII-tubulin) and olfactory fibroblast marker (Thy1.1), as assessed by immunocytochemistry. The overall objective was to develop a standardized method that yields reproducible outcomes. We investigated the effect of different bioprocess conditions, namely cell culture substrate, serum concentration, oxygen tension, enrichment with neurotrophic factor-3 (NT-3), and differential adhesion. Following this, we chose the most beneficial process conditions amongst the ones considered in this study, by which we could isolate pure OECs, and assessed their ability to support and promote neuronal growth in 2D neuron co-culture *in vitro*.

## Materials and Methods

### Cell isolation and culture

**Figure 1:**
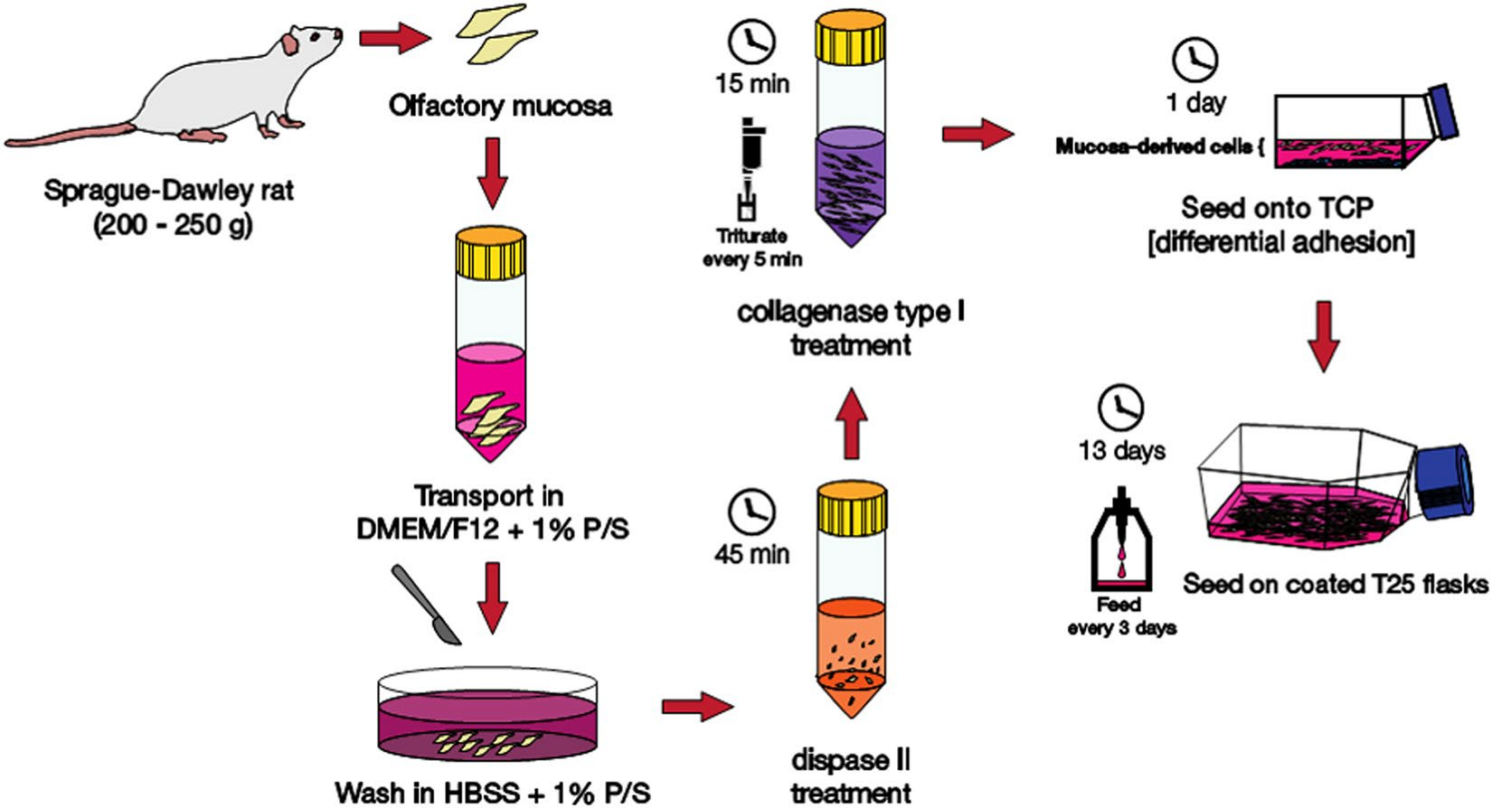
OEC isolation and culture from adult Sprague-Dawley female rats. This figure summarizes the best identified method of isolating OECs from rat primary tissue. It includes the initial dissection, the enzymatic digestion, the differential adhesion step as well as the full 14 days of the OEC culture (TCP = tissue culture plastic).

Rat nasal olfactory mucosa was collected from adult Sprague-Dawley female rats (200–250 g). This experiment was subject to local ethical review in accordance with the United Kingdom Animals (Scientific Procedures) Act 1986 (as amended), under establishment licence number X7069FDD2. Adult rats were euthanized by carbon dioxide asphyxiation (Schedule 1 method^28^) according to the UK Animals (Scientific Procedures) Act 1986. Following decapitation, the nasal septum was exposed and the olfactory mucosa was transferred to Dulbecco’s modified Eagles medium/Ham’s Nutrient Mixture (DMEM/F12) with GlutaMAX (Life Technologies) + 1% penicillin/streptomycin (P/S; Sigma-Aldrich UK). The mucosa was washed with Hank’s Balanced Salt Solution (HBSS) without calcium and magnesium (Life Technologies) + 1% P/S to remove excess mucus before being transferred to 6 cm dishes containing 2 or 10% fetal bovine serum (FBS) and cut into smaller 1 mm thick pieces with a scalpel. The mucosa was then transferred to and incubated in 2 ml of Dispase II (2.4 U/ml; Sigma-Aldrich UK) diluted in serum-free DMEM/F12 for 45 minutes at 37 °C, 5% CO_2_. Following this, the tissue was triturated and centrifuged at 400 × g for 5 minutes. The tissue and cell pellet were resuspended in 2 ml of 0.05% collagenase type I solution (Sigma-Aldrich UK) and incubated at 37 °C, 5% CO_2_ for 15 minutes, and triturated every 5 minutes. The resulting solution was centrifuged at 400 × g for 5 minutes and the cell pellet was resuspended in DMEM/F12 + 1% P/S + 2 or 10% FBS. The mucosae from three rats were pooled together for one experimental repeat (i.e. 3 pairs of mucosae from 3 rats) and seeded on to a T25 flask as seen in Fig. 1. Flasks were either not coated or coated with poly-D-lysine (PDL, 0.1 mg/ml; Sigma-Aldrich UK) or laminin (20 µg/ml; Millipore) depending on the experiment. The first media change was carried out on day 6 to allow time for the OECs to adhere; following this the media was changed every 3 days and cells were fixed at day 14 with 4% paraformaldehyde (PFA, Sigma-Aldrich UK) overnight at 4 °C. The cell isolation process is summarized in Fig. 1.

### Addition of neurotrophic factor-3

The cell isolation method is as described above with the addition of neurotrophic factor-3 (NT-3, 50 ng/ml, which was determined as optimum by Bianco *et al*.^29^) from day 6 onwards.

### *In vitro* co-culture with NG108-15 neurons

Co-culture experiments were carried out on PDL-coated glass coverslips (PDL being the best culture substrate for mucosa-derived OECs out of those tested in this study – PDL, laminin and TCP) in a 24-well plate. NG108-15 neurons were either grown alone, on a monolayer of mucosa-derived cells or on a monolayer of Schwann cells (SCL4.1/F7 Schwann cell line was obtained as frozen stocks from the Health Protection Agency; positive control). First 10^4^ mucosa-derived cells or Schwann cells were seeded onto the coated coverslip and allowed to adhere overnight, before 10^3^ NG108-15 neurons were seeded on the monolayer. The co-culture was fixed 5 days later with 4% PFA at 4 °C overnight. Media was changed every second day: DMEM/F12 + 1% P/S + 10% FBS.

### Immunocytochemistry

Following fixation of cells in 4% PFA overnight at 4 °C, the cells were washed (each wash step was carried out by dipping the coverslip in phosphate buffered saline solution (PBS, Sigma-Aldrich UK) 3 times). Subsequent steps were carried out at room temperature. Cells were incubated in 0.25% Triton-X solution (Sigma-Aldrich UK) for 20 minutes in order to permeabilize the membrane to allow for intracellular and surface marker staining. The aim of the permeabilization step is to ensure that a sufficient amount of p75NTR was detected for quantification. After another wash in PBS, the cells were incubated in a blocking solution containing 5% goat serum (DAKO UK) in PBS for 30 minutes. Following another wash, cells were incubated with primary antibody(s) at the appropriate dilution(s) in PBS for 90 minutes: 1:200 anti-neurotrophin receptor p75 (p75NTR, rabbit polyclonal; Millipore; and mouse monoclonal; Millipore), 1:200 anti-Thy1.1 (mouse monoclonal; Millipore), 1:200 anti-S100β (rabbit polyclonal, DAKO), 1:200 anti-glial fibrillary acid protein (GFAP, rabbit polyclonal; DAKO), 1:200 anti-nestin (mouse monoclonal; Millipore), and 1:200 anti-βIII-tubulin (mouse monoclonal; Sigma UK). Cells were washed and incubated with secondary antibody(s) at the appropriate dilution(s): 1:200 DyLight® 488 or 549, goat anti-rabbit IgG (H + L), or 1:200 DyLight® 488 or 549, goat anti-mouse IgG (H + L) (Vector Laboratories, US), and Hoechst 33258 (1:1000; Sigma-Aldrich UK) in PBS for 45 minutes. After a final wash, coverslips were mounted on slides using FluorSave™ reagent (Millipore).

Five images were taken per well in a cross formation around the center of the well. Two to three wells were stained per condition. After imaging, each channel was examined to assess whether the images taken were representative of the well. Brightness and shutter speed were set by identifying a cell that was determined to be positive and ensuring the background was dark to prevent overexposure of the cells. These settings were held constant between images for an experiment to ensure the images could be directly compared.

Images were analyzed using ImageJ. A color threshold was set to identify a positive cell, and only cells that were identified to be above this threshold were counted as positive. This prevented bias from entering the interpretation of results. Positive cells were counted and results were calculated as a proportion of cells positive for the marker and the yield of positive cells over the imaged area.

### Circularity analysis

The measurement of circularity can be used for enumeration of cell morphology traits. A macro was written in the open source Java image processing program ImageJ to automate the process of analyzing circularity. The threshold was adjusted for each individual image to ensure the best resolution was obtained. Cells with an area larger than 4000 pixels^2^ (2973 μm^2^) and smaller than 100 pixels^2^ (74 μm^2^) were discarded to prevent dense cell clusters and cell debris being included in the calculation. In addition to this, any cells on the image boundary were not counted. Circularity was calculated according to Equation 1, where *A* (μm^2^) is the area and *P* is the perimeter (μm). This was applied to S100β positive staining to determine the morphology of the cells.

1 $${\boldsymbol{Circularity}}=\frac{{\bf{4}}{\boldsymbol{A}}{\boldsymbol{\pi }}}{{{\boldsymbol{P}}}^{{\bf{2}}}}$$

### Neuronal growth analysis

Fluorescence microscopy (*EVOS FL*, *Life Technologies*) was used to capture images from immunostained coverslips. To assess neuronal outgrowth, all of the βIII-tubulin-positive neurons present in 233 833 µm^2^ (area of three images) per condition were manually traced using ImageJ 1.46r software to measure the length of the extension of each neurite. In addition, the number of neuronal cell bodies and the number of neuronal extensions were counted manually.

### Statistical analysis and data accessibility

All the results are presented as the average value from four (n = 4) independent experiments unless stated otherwise, and the error bars represent the standard error of the mean. Two-tailed Student’s *t-test* at a 95% confidence level or one-way ANOVA (95% confidence with Tukey’s post-test) were used to assess significant differences between independent samples where appropriate.

The datasets generated during and/or analyzed during the current study are available from the corresponding author on reasonable request.

## Results and Discussion

### Bioprocess modifications can increase OEC purity

**Figure 2:**
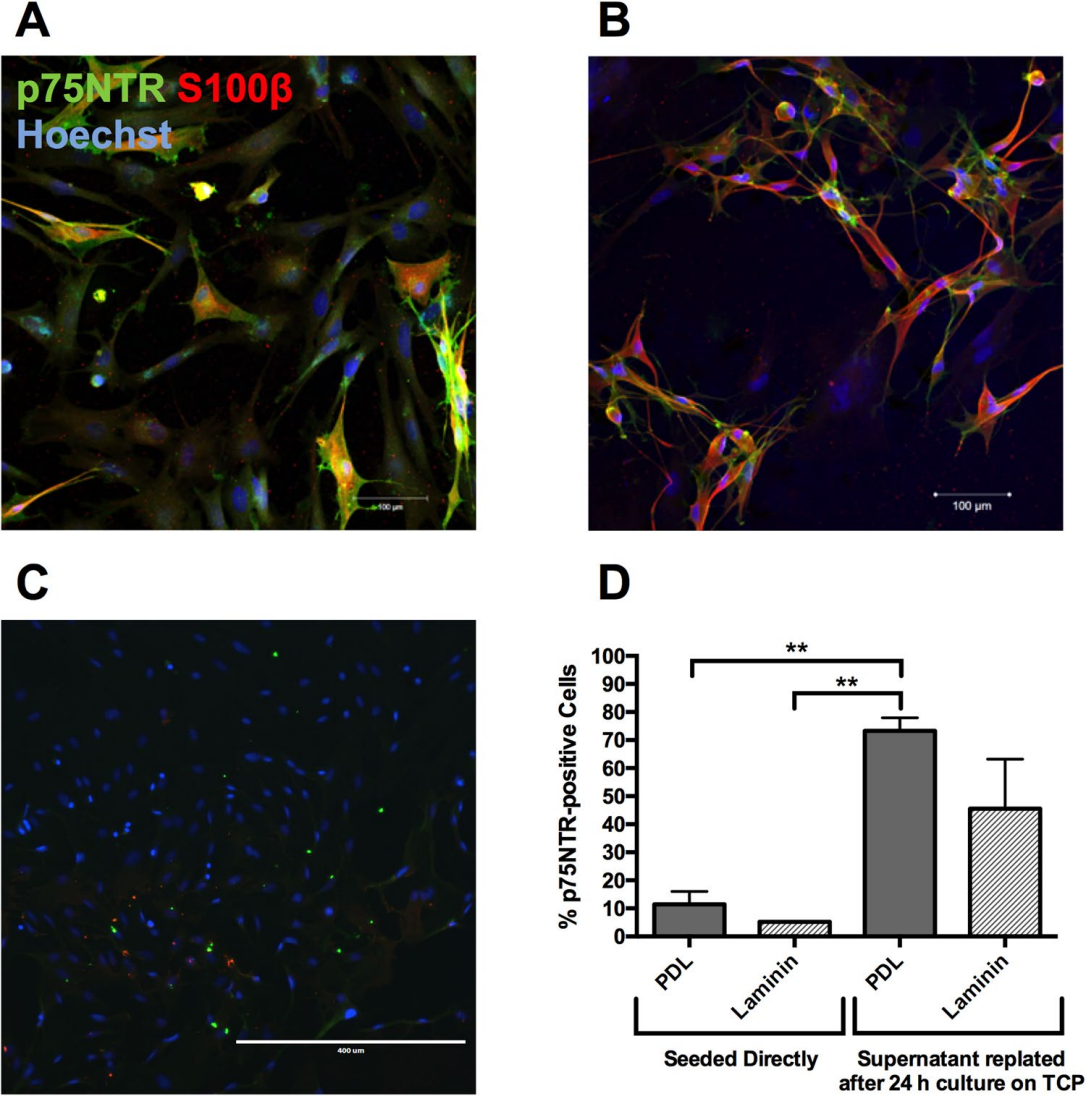
Differential Adhesion Step: Fluorescent micrographs of mucosa-derived OECs after the 24-hour differential adhesion on PDL (**A**), and Laminin (**B**). Scale bars are 100 µm. (**C**) Fluorescent micrographs of remaining adherent cells in the differential adhesion step. Following the cell isolation process, mucosa-derived cells were seeded either directly onto PDL or laminin for a 2-week culture period; or were first seeded on to TCP for 24 hours, before the supernatant was re-seeded onto PDL or laminin for the remainder of the 2-week culture period. All cells were cultured in DMEM/F12 + 1% P/S + 10% FBS. Mucosa-derived cells were fixed overnight at day 14 and stained to detect p75NTR protein. From these images, it can be seen that no glial cells were left behind due to the lack of positive glial cell staining (p75NTR, S100β) and the presence of fibroblastic markers Thy1.1 and α-SMA. Scale bars are 400 µm. (**D**) Purity of neurotrophin receptor p75NTR positive cells derived from the rat mucosa. **P < 0.05, one-way ANOVA with Tukey’s post-test. Data are mean values ± SEM, n = 3.

Firstly, we investigated explant culture where the mucosal tissue (cut in 1 mm thick pieces) was plated on PDL or laminin substrate and allowed to settle. After days of observation, no cells had migrated out the tissue, thus explant culture was discarded and enzymatic digestion was performed. Following cell isolation from rat mucosal tissue, cells were cultured on PDL or laminin matrices for 14 days; or cells were first seeded on to tissue culture plastic (TCP) for 24 hours before the supernatant was re-seeded on to PDL or laminin matrices. After 14 days in culture, 11 ± 5% of the mucosa-derived cells cultured directly on to PDL showed positive immunoreactivity for the OEC marker p75NTR, and 5 ± 0% of mucosa-derived cells cultured directly on laminin were positive for p75NTR as seen in Fig. 2A. We acknowledge that within the mucosa-derived population there might be a certain sub-population of Schwann cells that also express p75NTR; however, reviews of the literature indicate that there is a lack of putative biomarkers to clearly distinguish cell identity within mucosa-derived cells^30^. Some studies in the literature have reported p75NTR purities as high as 92% for bulb OECs^31^, which are intrinsically different from mucosa-derived OECs, such as those used in this work^32,33^. Additionally, it has been shown that mucosal OECs present a higher level of contaminant cell types due to the nature of the biopsy method^32,34^. Therefore, the differences in purity between mucosal and bulb OECs are inevitable. The purity of p75NTR-positive cells that were cultured directly on to the two matrices was significantly lower compared to the purity of p75NTR-positive cells cultured with a 24-hour differential adhesion step first on TCP and then PDL.

The purpose of the differential adhesion step was to remove the olfactory fibroblast cell impurities^25^, which adhere faster to the TCP surface than p75NTR-positive cells, which adhere more slowly (around 5 days) and thus increasing cell purity of p75NTR-positive cells by re-seeding the supernatant on another matrix. Following the differential adhesion step, 73 ± 5% and 46 ± 18% of the cell population were p75NTR- positive cultured on PDL and laminin, respectively, following a 24-hour differential adhesion step on TCP (Fig. 2A). No significant differences were found between PDL and laminin after the differential adhesion step as shown in Fig. 2A. Therefore, further experiments were carried out using both matrices in order to obtain a more informed comparison in different conditions. The purity was significantly higher for cells cultured on PDL after a 24-hour differential adhesion step, compared to cells cultured on laminin or PDL directly (*P* = 0.0040 and *P* = 0.0071 respectively, *P* < 0.05, one-way ANOVA with Tukey’s post-test).

To confirm that no OEC cells were lost in the differential adhesion step, the cells adhered in the first 24 hours were stained to detect glial cell markers (p75NTR and S100β) and fibroblast markers (Thy1.1 and α-SMA). Results provided evidence that no glial cells had adhered and were removed in the differential adhesion step and that the adhered cells were all positive for the fibroblast markers Thy1.1 and α-SMA, as can be seen in Fig. 2B.

Many other studies have looked at ways in to remove the inevitable fibroblasts present in the mucosa. This is an important step as any cell line heading for cell therapy needs to be completely characterized in order to guarantee the safety of the patient. If there are multiple cell types present in varying proportions, the function of the injected cells cannot be accurately predicted. Safety is the highest priority for any medicinal treatment. The key issue that exists for purifying OECs is the inherent plasticity of the population. Any purification method to separate OECs from other mucosal cells could inadvertently remove OECs that fulfil a different function to the rest of the population^35^. Other studies that carried out purification most commonly used antibody coated plates or beads to separate out the p75NTR positive cells or Thy1.1 negative cells in order to purify their sample up to ten days after the initial culture^22–24,36^. We believe that the method we have developed is more advantageous, as it uses no antibody, just a standard plastic tissue culture flask (Nunc^TM^ Cell Culture Treated Flask with Filter Cap), which is a cheaper alternative. This is important for eventual transfer to the clinic. It is also able to be carried out immediately, and time and material are not lost in culturing cells that are not needed. In addition to this, we did not observe any p75NTR-positive OECs that were left behind (Fig. 2B), thus we can be confident that the majority of the OEC population is carried forward to development and characterization. We also attempted to use 10 µM Ara-C (Sigma-Aldrich UK) over 24 hours from day 6 of culture, since Ara-C has been documented to kill fast-growing cells, such as fibroblasts^37–39^. However, we observed no enrichment of OECs (as seen in Supplementary Figure S1A,B) despite the apparent reduction in the number of fibroblasts.

Having confirmed that the inclusion of a 24-hour differential adhesion step immediately after cell isolation can increase purity by more than 6-fold, this step was carried forward for the isolation and culture of cells in subsequent experiments. Ideally, culture media would be well-defined, serum-free and possess the ability to maintain the regenerative phenotype of OECs. With this in mind, the effect of culture at a lower serum concentration on p75NTR-positive cell purity was assessed. In addition, reports in the literature demonstrated that the addition of neurotrophic factor-3 (NT-3) enriched the population for OECs^29^, and consequently we investigated the addition of NT-3 to media at low (2%) and high (10%) serum concentrations.

**Figure 3:**
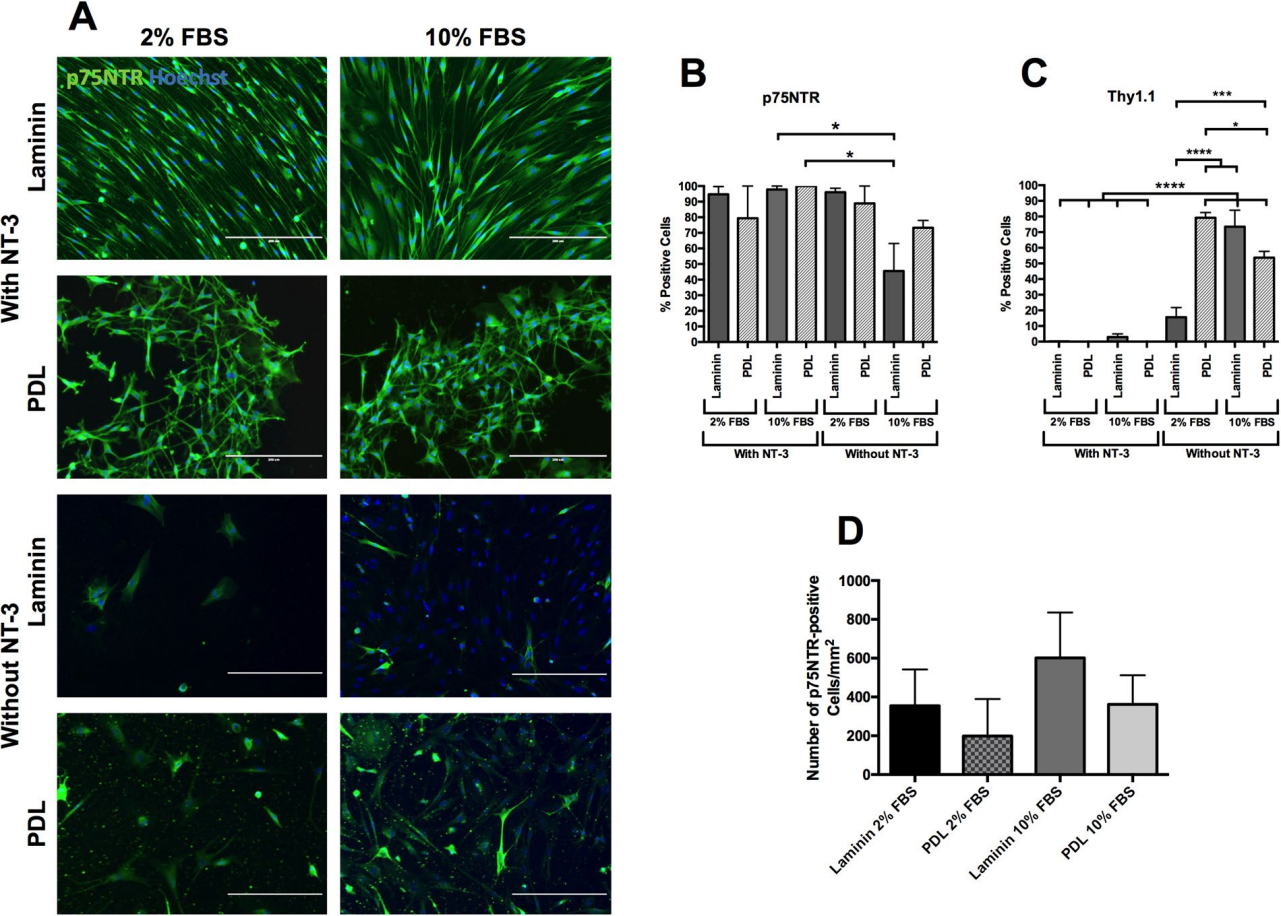
Effect of the presence/absence of neurotrophic factor-3 (NT-3) in 2 or 10% foetal bovine serum (FBS) conditions; (**A**) Fluorescent micrographs of mucosa-derived cells labelled with p75NTR. From these images it can be seen that the presence of NT-3 enhances the yield of p75NTR positive cells as well as encourages a spindle like morphology. Between the two different serum concentrations there was no noticeable change in yield. Scale bars are 200 µm. Cell purity of p75NTR (**B**) and Thy1.1 (**C**) of mucosa-derived cells cultured in the presence/absence of neurotrophic factor-3 (NT-3) in 2 or 10% foetal bovine serum (FBS). When cells were cultured on laminin with 10%FBS without NT-3, there are significantly (one-way ANOVA, Tukey’s post hoc test, P = 0.05) fewer p75NTR positive cells compared with laminin with 10% FBS and PDL with 10% FBS, both with NT-3 (**B**). These are the only conditions that reveal any significance. In terms of other coatings and media compositions, all of the other conditions show high percentages of p75NTR. In terms of Thy1.1 expression (**C**), more interactions are observed. All of the conditions without NT-3 with the exception of laminin with 2%FBS resulted in a significant (P < 0.0001) upregulation of Thy1.1 compared with conditions with NT-3. This indicates that without NT-3, the fibroblasts in the culture are able to proliferate faster than the OECs. From these results, it would appear that NT-3 is supporting the growth of OECs and therefore should be added to culture medium. (**D**) p75NTR yield of mucosa-derived cells cultured in the presence of neurotrophic factor-3 (NT-3) in 2 or 10% foetal bovine serum (FBS) conditions. The number of p75NTR-positive cells cultured in the presence of NT-3 were counted to determine the cell yield. There was a higher yield in 10% serum conditions on laminin (601 ± 117 cells/mm^2^) and PDL (362 ± 75 cells/mm^2^), compared with 2% serum cultures, although none of the groups were significantly different (one-way ANOVA). Data are means ± SEM, *n = *4.

Two FBS serum concentrations were investigated: 2 and 10%, and OEC purity was assessed after 14 days in culture on PDL or laminin matrices in media with/without NT-3 (50 ng/ml). At day 14, cells were fixed and stained to detect p75NTR and Thy1.1. The fluorescent micrographs shown in Fig. 3A indicate that there were more cells present in 10% serum cultures compared to cells cultured in 2% serum. Cultures with the addition of NT-3 had a much higher proportion of cells that were p75NTR-positive, compared with cultures that were not supplemented with NT-3. The morphology of the cells in the presence of NT-3 was glial-like, where the majority of cells were elongated and spindle-shaped. This was especially evident for cells cultured on laminin. This was in contrast to the p75NTR-positive cells cultured in the absence of NT-3 that had a fibroblast-like cell shape, although there were some cells present with an elongated morphology (Fig. 3A).

The purity of OECs was determined by counting the immunofluorescently labelled p75NTR-positive cells, and the percentage of fibroblast impurities was also assessed by counting the number of Thy1.1-positive cells, a marker for rat olfactory fibroblasts. The results are shown in Fig. 3B,C.

Quantification of the fluorescent micrographs is shown in Fig. 3B–D. From this data, it can be seen that the purity of p75NTR (Fig. 3B) remained reasonably high (up to 100%) for most conditions. The two conditions that suffered lower purities, were laminin and PDL with 10% FBS and no NT-3. This difference was significant (*P* < 0.05, one-way ANOVA with Tukey’s post-test) between PDL, 10% FBS, no NT-3 and PDL and laminin with 10% FBS with NT-3. From these results, it can be determined that NT-3 is having a significant impact on p75NTR expression in 10% FBS conditions.

When the purity of Thy1.1 (Fig. 3C) is considered, the absence of NT-3 also has a significant impact on protein expression. In this case, when NT-3 is removed from the media, Thy1.1 is significantly (P < 0.0001, one way ANOVA with Tukey’s post-test) upregulated. Combined with what is observed in Fig. 3B, this would indicate that NT-3 and serum concentrations are both critically important for OEC selection. When serum levels are high (10%) and NT-3 is absent, the media components select for the fibroblast impurities, which results in a significant upregulation. When NT-3 is present and serum levels are lower (2%), the OECs were more abundant that fibroblasts (Fig. 3B,C). The higher serum concentration appears to have a more significant effect on the faster dividing fibroblasts than it does on the OECs. This may be why lower p75NTR and higher Thy1.1 expression is seen at higher serum conditions. The presence of NT-3 seems to give the rat OECs an advantage over the fibroblasts as in conditions with NT-3, there is less than 5% Thy1.1 expression, in most cases, close to 0%.

When the yield is considered (Fig. 3D) for cells only cultured in NT-3, laminin with 10% FBS appears to give the highest p75NTR yield (601 ± 117 cells/mm^2^); however, no significance was found (one-way ANOVA). As discovered in the purity quantification (Fig. 3B,C), care needs to be taken with 10% FBS, as this can allow the fibroblast impurities to benefit more than the OECs.

From the variables examined in this study, the optimum culturing conditions for primary rat OECs have been identified as a concentration of 2% FBS and a concentration of 50 ng/ml NT-3 on laminin coating. This allows for a high purity of p75NTR cells to be attained while minimizing the presence of Thy1.1 positive fibroblasts. By maximizing the purity of the desired OEC cell type, this ensures any subsequent purification steps become easier as the initial level of contaminants is lower.

There have been few studies carried out on the appropriate flask coating for OECs. It is recognized that OECs attach to laminin. Therefore, it follows that OECs would prefer this coating^40^. Despite this, most research groups have carried out OEC culture on PLL coated flasks or cover slips^27,31,41,42^. PLL (an isomer of PDL) is normally used to aid the adhesion of neuronal cells to culture plastic^43^. Laminin on the other hand is typically used for the culture of stem cells, as it has been observed that laminin interacts with integrins or other components of the cell matrix to inhibit differentiation^44^. This may explain why previous research has used PLL successfully without exploring other options. Ingram *et al*. (2016) compared the use of PLL to laminin in regards to the migratory properties of OECs and found using transwells that twice as many OECs migrated through laminin coated transwells compared to PLL^45^. Although our work did not examine migration, the preference OECs have for laminin is the same. The higher purity may be in part due to fewer fibroblasts attaching and proliferating, and studies have shown in the past that fibroblasts prefer fibronectin as a substrate and struggle with fast proliferation on laminin^46^.

In summary, there were fewer or no fibroblasts present in all cultures with NT-3 compared to all cultures without NT-3. The purity of p75NTR-positive cells, a putative OEC marker, was high in all cultures with NT-3 and cultures without NT-3 in 2% serum. These data indicate that the culture conditions, 2% serum, in the presence of NT-3 (50 ng/ml) on a laminin matrix, are desirable to maximize the purity of the desired OEC cell type making subsequent purification steps easier.

### Characterization of mucosa-derived cell populations at normal (atmospheric) and low (physiological) oxygen

Next, we investigated the effect of physiological oxygen tension on the culture of rat mucosa-derived cells. We compared a physiological oxygen tension (2–8%) to the typically used 21% oxygen. Cells were isolated as described in the Materials and Methods section, including a differential adhesion step, and then cultured on laminin in 2% serum and supplemented with NT-3 (50 ng/ml). Cells were fixed at day 14 and stained to detect: putative OEC marker, p75NTR; glial cell markers, S100β and GFAP; neuronal precursor cell markers, nestin and βIII-tubulin; and olfactory fibroblast marker, Thy1.1.

**Figure 4:**
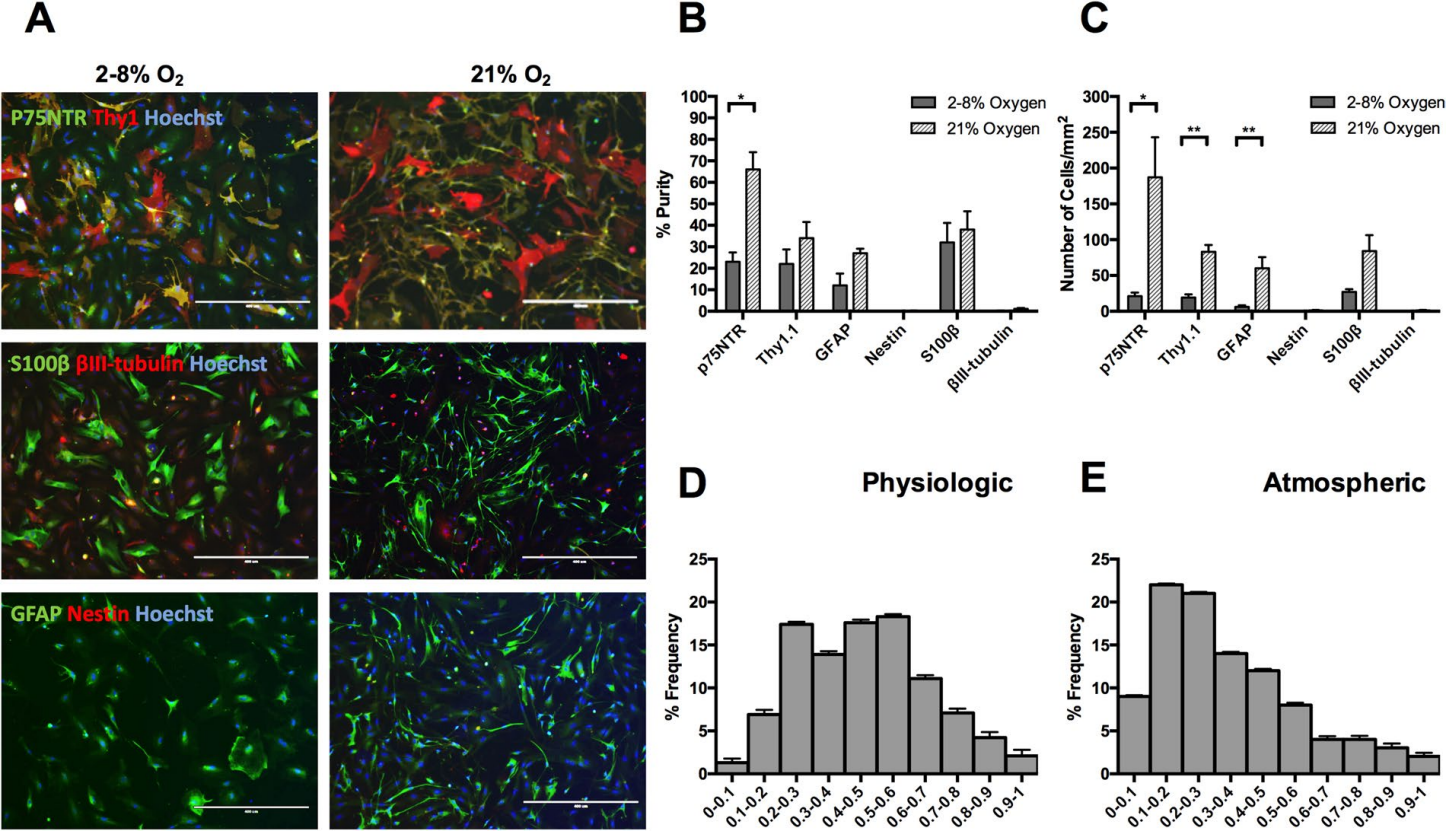
Characterisation of mucosa-derived cell populations cultured at physiological or atmospheric oxygen (O_2_) concentrations; (**A**) Fluorescent micrographs Following cell isolation, cells were cultured at either physiological O_2_ (2–8%) or atmospheric O_2_ (21%), on laminin + 2% serum + NT-3 (50 ng/ml). At day 14, cells were fixed and stained for p75NTR, S100β (peripheral nerve glial marker), Thy1.1, nestin (a marker for neuronal precursor cells), glial fibrillary acidic protein (GFAP, a marker for CNS glia) and βIII-tubulin (a neuronal marker). Scale bars are 400 µm. (**B**) Purity of mucosa-derived cell populations cultured at physiological or atmospheric oxygen (O_2_) concentrations. Quantification of the fluorescent micrographs revealed that cells were mostly positive for p75NTR, Thy1.1, S100β and GFAP, and the proportions of cells positive for these markers was higher at 21% oxygen. However, this difference was only significant for p75NTR immunoreactivity (*P = 0.0105, unpaired t-test). (**C**) Yield of mucosa-derived cell populations cultured at physiological or atmospheric oxygen (O_2_) concentrations. There was a higher yield of mucosa-derived cells cultured in 21% O_2_. There was a significantly higher yield of cells positive for p75NTR, Thy1.1 and GFAP, with *P* values: *P = 0.039, **P = 0.0038, **P = 0.0097, respectively (unpaired t-test). Data are means ± SEM, *n* = 4. Circularity of mucosa-derived cell populations cultured at physiological (**D**) or atmospheric (**E**) oxygen (O_2_) concentrations. Data represents the mean values ± SEM, *n* = 19

Fluorescent micrographs show that there were more cells present at 21% oxygen culture than at 2–8% oxygen culture (Fig. 4A). There were a higher proportion of cells positive for the glial cell markers (p75NTR, S100β and GFAP) at 21% oxygen compared to 2–8% oxygen. Furthermore, cells at both 2–8% and 21% oxygen were negative for neuronal precursor markers (nestin and βIII-tubulin; Fig. 4A). This is further confirmed by quantification in Fig. 4B,C.

The cell morphology was quantified using the measurement of circularity. A circularity of one indicates a perfect circle and a circularity of zero indicates a straight line (i.e. an elongated cell). It was found that when cells were cultured under normal oxygen conditions the distribution was shifted to the left as seen in Fig. 4E. This indicates more spindle shaped cells compared to physiological oxygen conditions where the distribution was more centralized, representing rounder shaped cells (Fig. 4D,E). The circularity distribution is important since cell morphology is related to functionality; the spindle-shaped morphology has been defined in the literature as exhibiting the desired regenerative properties *in vivo* for OECs^30,41,47,48^. These observations are backed up by the skewness values for the distributions. The normal oxygen distribution had a skew value of 0.9 which characterizes its shift to the left. The smaller shift of the low oxygen histogram is reflected in its smaller skewness value of 0.2.

Cells were counted to determine the purity and yield of the different cell phenotypes present in the mucosa-derived cell populations (Fig. 4B,C). The proportion of p75NTR-positive cells was less than we observed previously for cells cultured in the same conditions: with NT-3 in 2% serum and on a laminin matrix. This is due to the natural biologic variability of OECs from mucosae isolated from different animals^49^. The proportion of olfactory fibroblasts was also higher here (Fig. 4B,C).

Cells cultured at 21% oxygen had a significantly higher proportion of cells positive for p75NTR (66 ± 9%) than the 2–8% oxygen culture (23 ± 4%; *P* < 0.05, unpaired t-test). Although there were a higher proportion of cells positive for Thy1.1, GFAP, and S100β in 21% oxygen culture, compared to 2–8% oxygen, these differences were not significant (Fig. 4B). None of the cells were positive for nestin at 2–8% oxygen, which was similar to the proportion of nestin-positive cells at 21% oxygen (0.3 ± 0.3%). Similarly, less than 1% of cells were positive for βIII-tubulin at both 2–8% and 21% (Fig. 4B).

Although the purity of Thy1.1-positive cells was not significantly different at 2–8% and 21% oxygen, the yield of Thy1.1-positive cells (83 ± 10 cells/mm^2^) was significantly higher at 21% oxygen than at 2–8% oxygen (19 ± 5 cells/mm^2^; *P* < 0.01, unpaired t-test). Quantification revealed that there were 187 ± 56 cells/mm^2^ yield of p75NTR-positive at 21% oxygen, which was significantly higher than 21 ± 5 cells/mm^2^ at 2–8% oxygen. The yield of S100β-positive cells was also higher at 21% oxygen than at physiological oxygen but this was not significant. Almost none of the cells were positive for the neuronal precursor markers nestin and βIII-tubulin (Fig. 4B,C).

The fact that there were significantly more cells at 21% oxygen is not wholly unexpected. Although the majority of the cells in the body experience physiological oxygen conditions, the cells in the mucosa are in the lining of the nose and therefore are used to higher oxygen levels. When the oxygen tension is lowered (the average in the CNS is 3%), these cells are essentially being starved of oxygen and therefore a reduction in the yield and purity of these cells can be observed^50^. This is an issue that needs further research as the cells in the spinal cord do not experience high levels of oxygen and OECs need to be able to function at this oxygen tension if they are expected to aid neural regeneration and recovery.

The results obtained using all the conditions described so far are summarized in Table 1. The effects that those conditions have on p75NTR and Thy1.1 purity are shown in a simplified manner. Generally, an increase in p75NTR purity is observed as a positive effect (+), whereas an increase in Thy1.1 purity is considered a negative effect (−).

**Table 1:** Summary of the effects of the different conditions on p75NTR and Thy1.1 purity.

Condition	Purity (%, p75NTR)	Purity (%, Thy1.1)	Effect	Additional Information
Differential Adhesion Laminin	46	N/A	+	Without NT-3 10% FBS 21% Oxygen
Differential Adhesion PDL	73	N/A	++	Without NT-3 10% FBS 21% Oxygen
No differential Adhesion Laminin	5	N/A	−	Without NT-3 10% FBS 21% Oxygen
No differential Adhesion PDL	11	N/A	−	Without NT-3 10% FBS 21% Oxygen
**Laminin with NT-3 2% FBS**	**95**	**0**	**+++**	**21% Oxygen**
PDL with NT-3 2% FBS	80	0	+	21% Oxygen
Laminin with NT-3 10% FBS	98	3	++	21% Oxygen
PDL with NT-3 10% FBS	100	0	++	21% Oxygen
Laminin without NT-3 2% FBS	96	16	+	21% Oxygen
PDL without NT-3 2% FBS	89	79	−	21% Oxygen
Laminin without NT-3 10% FBS	46	73	−	21% Oxygen
PDL without NT-3 10% FBS	73	54	−	21% Oxygen
21% Oxygen	66	34	+	Laminin with NT-3 2% FBS (optimal condition)
2–8% Oxygen	23	22	−	Laminin with NT-3 2% FBS (optimal condition)

+++ represents the optimum condition (also shown in bold), ++ represents a very positive effect, + represents a positive effect, − represents a negative effect, and–represents a very negative effect. An increase in p75NTR purity is observed as a positive effect (+), whereas an increase in Thy1.1 purity is considered a negative effect (−).

### Co-culture with neurons and compared to F7 Schwann cells

The effect of rat olfactory mucosa-derived cells co-culture with NG108-15 neuronal cell line with comparison with NG108-15 cell culture on PDL negative control or on F7 Schwann cells (F7/SC) positive control was investigated. Cells were isolated as described in the Materials and Methods, including a differential adhesion step, and then cultured on laminin in 2% serum at atmospheric oxygen and supplemented with NT-3 (50 ng/ml) at day 6. Cells were co-cultured for three or five days at atmospheric and physiological oxygen and stained to detect S100β glial cell marker and βIII-tubulin neural cell marker.

The number of cells, extensions and their length in the NG108-15 cell population was determined for the three cultures: NG108-15 neurons on PDL, NG108-15 on F7/SC and NG108-15 on rat OECs at three and five days at both physiologic and atmospheric oxygen.

**Figure 5:**
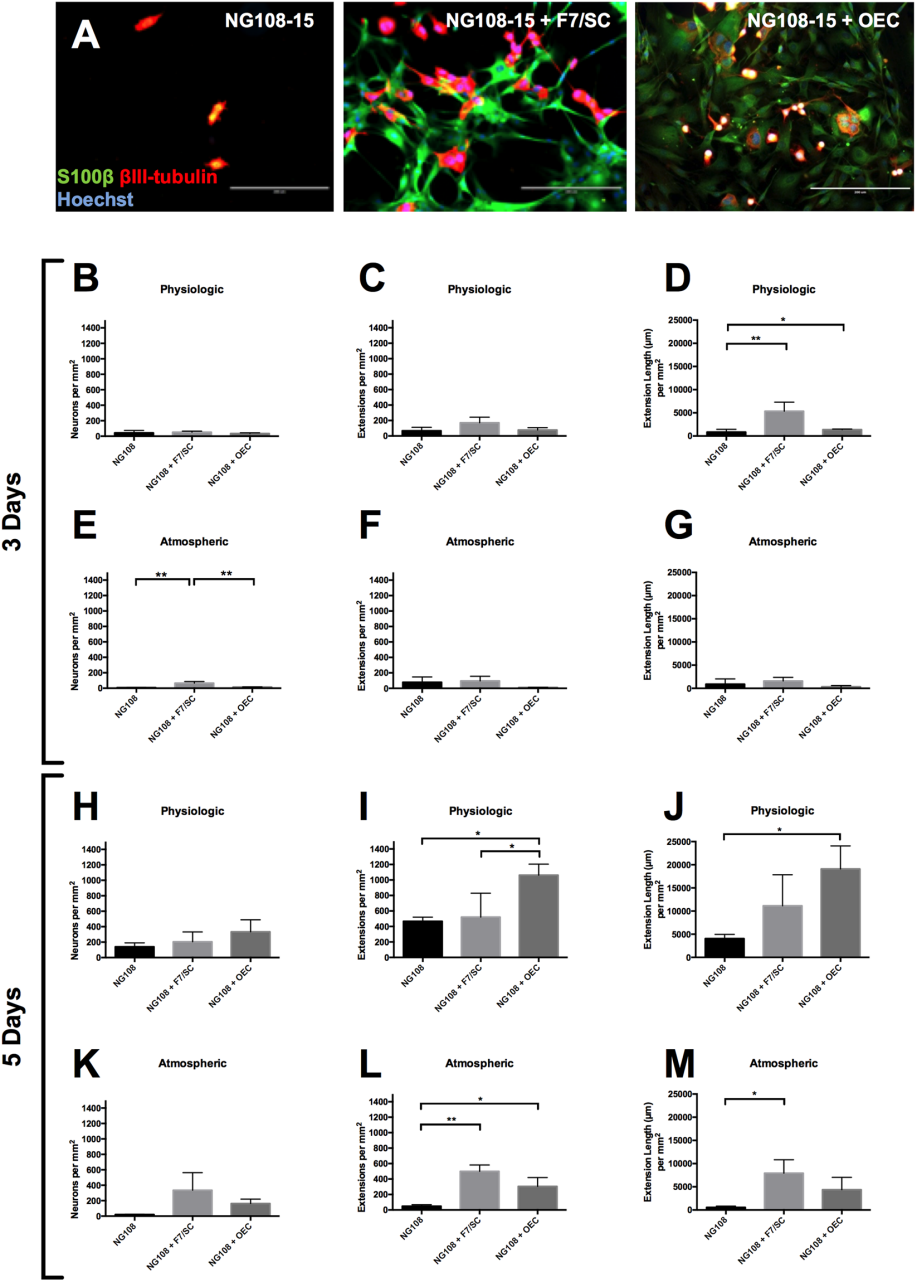
Co-culture of NG108-15 neuronal cell line with rat olfactory mucosa-derived cell populations for 5 days at atmospheric oxygen (**A**); NG108-15 behavior on rat OECs, F7 Schwann cells and PDL for 3 days (**B**–**G**) and 5 days (**H**–**M**). (**A**) Following cell isolation, cells were cultured at atmospheric oxygen (21%), on laminin + 2% serum + NT-3 (50 ng/mL). At day 14, NG108-15 cells were cultured with rat OECs, F7 Schwann cell line or on PDL. After a further 5 days in culture, cells were fixed and stained for S100β (peripheral nerve glial marker), βIII-tubulin (a neuronal marker) and Hoechst. These fluorescent micrographs suggest a higher neuronal cell number with more and longer extensions for co-culture with rat OECs or F7/SC, compared with culture on PDL. Scale bars are 200 µm. (**B**–**M**) ANOVA, 95% confidence, was carried out on the data shown. Data are means ± SEM, *n* = 3.

The co-culture (5 days) fluorescent micrographs can be seen in Fig. 5A, showing a higher number of neurons and extensions when co-cultured with F7/SC and OECs when compared to NG108-15 alone. It is also evident that a higher total extension length in the co-culture of NG108-15 with F7/SC and OECs was achieved.

For the three-day co-culture at physiologic oxygen, it can be seen that NG108-15 neurons with F7/SC had a very similar number of neurons compared with culture on PDL or co-culture with OECs, as seen in Fig. 5B. The same pattern was observed for the number of extensions, where neurons co-cultured with F7/SC had the highest number of extensions per mm^2^ when compared with co-culture on OECs or culture on PDL (Fig. 5C). For the extension length per mm^2^, co-culture with both F7/SC and OECs had a significantly higher value when compared with culture of NG108-15 neurons on PDL (P = 0.0083 and P = 0.0149 respectively, as observed in Fig. 5D).

At atmospheric oxygen, co-culture for three days with F7/SC had significantly higher number of neurons per mm^2^ when compared with co-culture with OECs (P = 0.0059), as seen in Fig. 5E. The number of neurons per mm^2^ was also lower with culture on PDL (P = 0.0028) when compared with co-culture with F7/SC and lower than co-culture with OECs, although not significantly (Fig. 5E). Co-culture with F7/SC had the highest number of extensions per mm^2^, although not significantly different from co-culture with OECs or on PDL (Fig. 5F). The longest extension length was observed for the co-culture with F7/SC, although not significantly different from co-culture with OECs or culture on PDL (Fig. 5G).

Due to the low number of neurons and neuron extensions shown in Fig. 5B–G, it was established that the culture time was not sufficient to draw definite conclusions, and it was decided to extend the co-culture time to five days.

Five days co-culture at physiological oxygen had the highest number of neurons per mm^2^ with co-culture with OECs, however it was not significantly different from co-culture with F7/SC or culture on PDL (Fig. 5H). Similarly, the number of extensions and extension length per mm^2^ co-culture with OECs had a significantly higher value when compared to co-culture with F7/SC and culture on PDL (positive and negative controls) as seen in Fig. 5I,J.

Regarding the experiments conducted at atmospheric oxygen (five-day co-culture), the number of extensions was significantly higher for the co-culture of NG108-15 with F7/SC when compared with NG108-15 culture on PDL (P = 0.0013), and the co-culture of NG108-15 with OECs also yielded a higher number of extensions than the negative control (P = 0.0210). The number of extensions for the co-culture of NG108-15 with OECs, although lower, was not significantly different from co-culture with F7/SC (Fig. 5L).

The co-culture of NG108-15 with F7/SC had significantly higher extension length when compared to NG108-15 co-culture on PDL. Even though the extension length of NG108-15 neurons in co-culture with OECs was higher when compared with NG108-15 on PDL, it was not statistically significantly different. It was also not significantly different from the co-culture with F7/SC (Fig. 5M).

The results shown in Fig. 5 provide evidence that the co-culture time of three days was not sufficient to discern whether the presence of OECs had a positive effect on neuronal development in terms of number of neurons, number of extensions and extension length. On the other hand, the extended co-culture (five days) at both physiological and atmospheric oxygen conditions did show promising results.

Co-culture of NG108-15 neurons with OECs at atmospheric oxygen conditions after five days of culture showed increased number of neurons and number of extensions when compared to neurons alone. Statistical differences were also seen between the positive and negative controls, providing reliability to the results of interest in this paper.

Interestingly, physiologic oxygen greatly increased NG108-15 neuronal development for the co-culture for 5 days. This might be related to the effect of low oxygen tension on neuronal stem cells and neuronal progenitors. NG108-15 cell line is a hybrid formed by Sendai virus-induced fusion of the mouse neuroblastoma clone N18TG-2 and the rat glioma clone C6 BV-1. Therefore, it is expected that these cells exhibit behaviour similar to neuronal progenitors or stem cells.

It has been shown that mild hypoxia (2.5 and 5% oxygen) increases neural stem cell (NSC) proliferation and neuronal and oligodendroglial differentiation^51^. Additionally, low oxygen (4% oxygen) concentrations may be involved in expansion of early NSC populations by inhibiting cell death through different pathways in sequential primitive NSC and definitive NSC populations.

Moreover, PC12 cell line derived from rat pheochromocytoma, with embryological origin similarly to neuroblastic cells, can easily differentiate into neuron-like cells^52^. When cultured in reduced oxygen (1%, 4%, or 12% oxygen) they exhibited significant increases in neurite extension and total neurite length when compared to atmospheric conditions^53^.

As expected, OECs promoted neuronal development in the form of increased number of neurons, extensions and extension length. Although improved neurite outgrowth has been observed before with contact of OECs with neurons, most of the tests *in vitro* were performed with OECs isolated from the bulb. In most of the studies this positive effect was visible between 5–17 days as was seen in our work, suggesting that the positive effect OECs have on neurons is time dependent^11,15,54–60^.

## Conclusions

We have established a method that increases the purity of primary mucosa-derived rat olfactory ensheathing cells by introducing a differential adhesion step, using NT-3 and culturing the cells at 21% O_2_ in the presence of 2% FBS. All of these steps result in the upregulation of key marker p75NTR and the downregulation of fibroblast marker Thy1.1. In addition, co-culture with a neuronal cell line determined that OECs cultured under the conditions stated, had significant improvement of neuronal development compared to neurons cultured on their own. However, there are still inconsistencies in yields and purities obtained across independent experiments and the source of variability is presently not known. Therefore, much work is still needed in order to create standardized and consistent isolation methods.

## Electronic supplementary material

Figure S1
