# Supplementary material for: Bioprocessing strategies to enhance the challenging isolation of neuro-regenerative cells from olfactory mucosa
Source: Sci Rep. 2018 Sep 27;8:14440. doi: 10.1038/s41598-018-32748-w (PMC6160430; doi:10.1038/s41598-018-32748-w)
Supplement: Supplementary file 1 — Figure S1 [file 41598_2018_32748_MOESM1_ESM.docx]

**Bioprocessing strategies to enhance the challenging isolation of neuro-regenerative cells from olfactory mucosa**

**Melanie Georgiou ^a b^, Joana Neves dos Reis ^a^, Rachael Wood ^a c^, Patricia Perez Esteban ^a c^, Victoria Roberton ^a^, Chris Mason ^a^, Daqing Li ^d^, Ying Li ^d^, David Choi ^d e^, Ivan Wall ^a c*^**

*^a^ Department of Biochemical Engineering, University College London, Torrington Place, London, WC1E 7JE, UK*

*^b^ Present address: Cell and Gene Therapy Catapult, Guy's Hospital, Great Maze Pond, London SE1 9RT, UK*

*^c^ Aston Medical Research Institute and School of Life & Health Sciences, Aston University, Aston Triangle, Birmingham, B4 7ET, UK*

*^d^ Spinal Repair Unit, Department of Brain, Repair and Rehabilitation, Institute of Neurology, University College London, Queen Square, London, WC1N 3BG, UK*

*^e^ National Hospital for Neurology and Neurosurgery, Queen Square, London, WC1N 3BG, UK*

* Author to whom correspondence should be addressed: Ivan Wall (i.wall@aston.ac.uk)


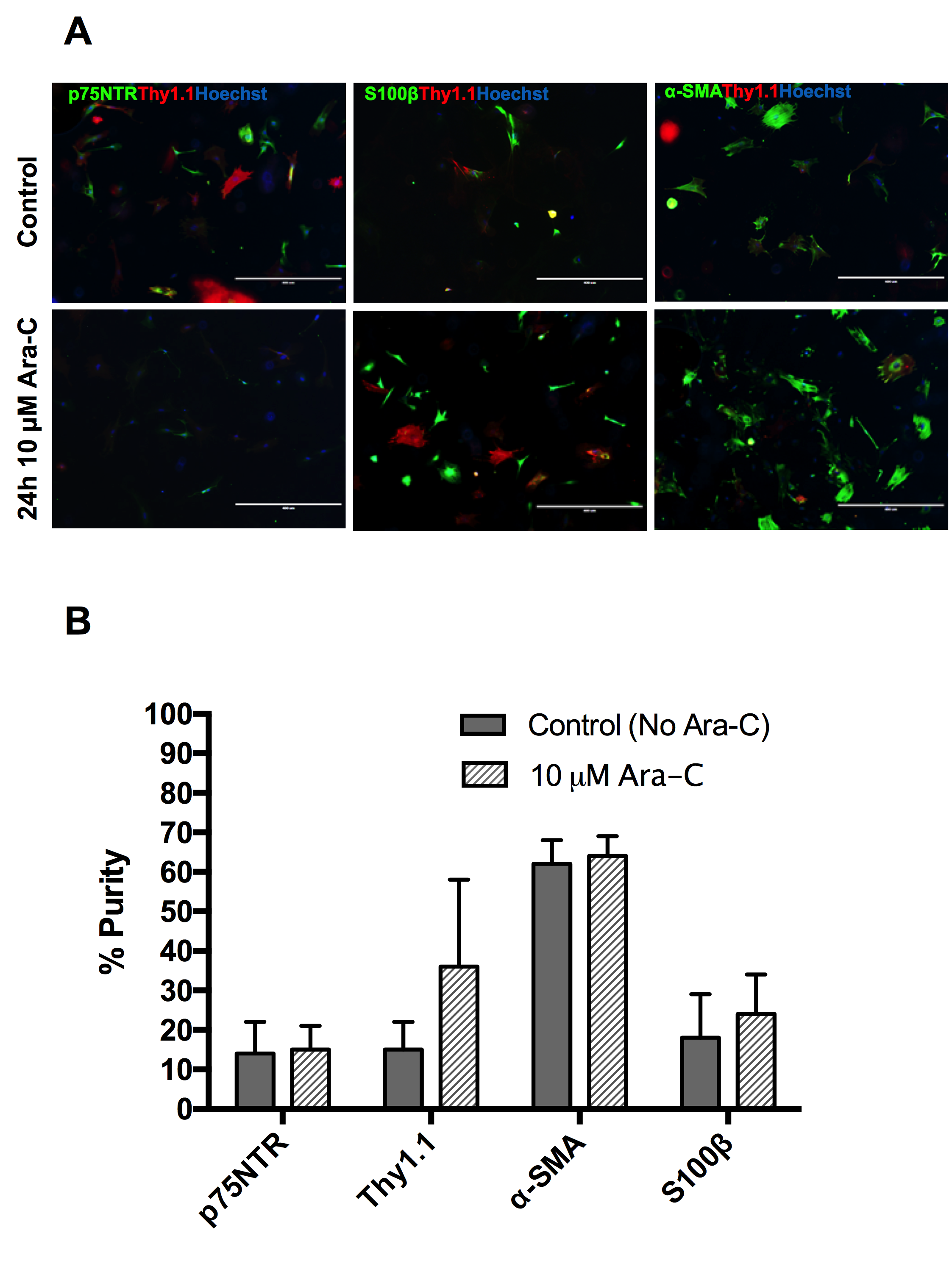


**Figure S1:** Impact of Ara-C on selective killing of fast-growing cell types. (A) Cells exposed to Ara-C for 24 hours, after 6 days of culture did not appear to be affected by Ara-C. (B) Measurement of percentage purity for p75NTR, Thy1.1, α-SMA and S100β confirmed this lac of effect. Data are means ± SEM, *n* = 3
